# Rs10887800 renalase gene polymorphism is associated with an increased risk of coronary artery disease in hemodialyzed patients

**DOI:** 10.1007/s11255-016-1270-7

**Published:** 2016-03-29

**Authors:** Anna Stec, Andrzej Ksiazek, Monika Buraczynska

**Affiliations:** Department of Nephrology, Medical University of Lublin, Jaczewskiego Street 8, 20954 Lublin, Poland

**Keywords:** Renalase gene, End-stage renal disease, Hemodialysis, Coronary artery disease

## Abstract

**Purpose:**

Coronary artery disease (CAD) is common in patients with end-stage renal disease (ESRD). Recent studies have suggested that renalase, a novel FAD-dependent amine oxidase, may play an important role in the pathogenesis of cardiovascular complications in ESRD patients. The aim of the study was to investigate the association between renalase gene polymorphisms and a risk of CAD in patients on hemodialysis.

**Methods:**

In a case–control study, a total of 309 hemodialyzed patients (107 with and 202 without CAD) were genotyped for two SNPs in the renalase gene (rs10887800 and rs2576178) using the PCR–RFLP method.

**Results:**

By multivariate logistic regression analysis, we found that rs10887800GG genotype was associated with an increased risk of CAD under the codominant model [GG vs AA; adjusted OR 2.66 (95 % CI, 1.19–5.94), *p* = .017] and under the recessive model [GG vs AG + AA; adjusted OR 2.10 (95 % CI, 1.10–4.02), *p* = .025]. The rs2576178 polymorphism did not influence the risk of CAD.

**Conclusion:**

The study suggested for the first time that the rs10887800 renalase gene polymorphism may be involved in the pathogenesis of CAD in hemodialyzed patients and thus could be considered a new genetic risk factor for CAD in this population.

## Introduction

Cardiovascular disorder, particularly atherosclerotic coronary artery disease (CAD), is the leading cause of morbidity and mortality in end-stage renal disease (ESRD) patients on maintenance hemodialysis [1]. Both prevalence and annual mortality for CAD are much higher in hemodialyzed patients compared to the age-matched general population (40 vs 7.3 % and 9 vs .28 %, respectively) [2]. The above data support the hypothesis that pathogenesis and manifestation of CAD differ in the presence of renal failure. CAD is a multifactorial, polygenic disease with environmental and genetic determinants. The accumulation of traditional cardiovascular predictors does not fully explain an excess risk of CAD in hemodialyzed patients [3]. The evaluation of novel risk factors, predominantly genetic markers, is thus essential to improve CAD risk stratification in patients with ESRD. Over the years, the candidate gene approach and genome-wide association studies have successfully identified CAD susceptibility genes in a general population. However, the number of such studies in ESRD patients remains limited [4, 5].

Renalase is a recently discovered flavin adenine dinucleotide (FAD)-dependent amine oxidase, not only expressed in kidneys, but also observed in cardiomyocytes, the nervous system and endothelium [6]. It degrades circulating catecholamines and thus regulates blood pressure and cardiac function [7]. It was suggested that renalase may play a key role in the pathogenesis of cardiovascular complications among patients with ESRD [8].

Renalase gene (RNLS) resides on chromosome 10 (10q23.31), has 13 exons and encodes a 342 amino acids protein with a calculated molecular mass of ~38 kDa. Recent studies have explored the association between variants in the renalase gene and an increased risk of cardiovascular disorders [9, 10].

The aim of the present study was to investigate the association between rs10887800 and rs2576178 renalase gene polymorphisms and a risk of CAD in hemodialyzed patients.

## Materials and methods

### Subjects

The case–control study population comprised 309 patients with end-stage renal disease (ESRD) undergoing hemodialysis, consecutively enrolled between January 2012 and December 2013. Overall, there were 157 males (50.8 %) and 152 females (49.2 %). Mean age was 64.1 ± 14.1 years. ESRD resulted from diabetic nephropathy (23 %), chronic glomerulonephritis (18.2 %), hypertensive nephropathy (12.6 %), polycystic kidney disease (10 %), interstitial nephritis (9.1 %), obstructive nephropathy (8.4 %) and other or unknown causes (18.7 %). A positive family history of CAD in first-degree relatives was reported by 42 patients (13.6 %).

All recruited patients underwent HD procedure three times a week—3 to 5 h each at five dialysis centers in eastern Poland. Blood samples for genetic studies were taken into EDTA tubes from the venous part of the dialysis access at the beginning of HD. All HD patients underwent 12-lead resting electrocardiography (ECG). Baseline demographic and clinical data (including signs and symptoms of CAD) were obtained from a standardized interview and a physical examination, both provided by a specialist from the Department of Nephrology in Lublin. The clinical data were complemented by the analysis of medical records. Patients enrolled in the study were divided into two subgroups according to the presence of coronary artery disease (CAD): the CAD+ group consisted of 107 patients with CAD and the CAD− group consisted of 202 patients without CAD.

The CAD diagnosis was based on the criteria of the European Society of Cardiology (ESC).

Patients were enrolled to the CAD+ group if they met at least one of the following inclusion criteria: (1) history of CAD, myocardial infarction or angina pectoris, (2) previous percutaneous coronary intervention or coronary artery bypass surgery, (3) abnormal coronary angiography or CT angiography result.

Patients with other cardiac diseases such as hypertrophic or dilated cardiomyopathy, aortic stenosis or pericarditis were excluded from the study.

### Genotype determination

Genomic DNA was isolated from peripheral blood leukocytes according to the technique of Madisen et al. [11] with minor modifications.

Genotypes for two SNPs in the renalase gene (dbSNP Accession Numbers: rs2576178 and rs10887800) were determined by the polymerase chain reaction–restriction fragment length polymorphism (PCR–RFLP) method. The following primers were used for amplification: for rs2576178 polymorphism forward 5′-AGCAGAGAAGCAGCTTAACCT-3′ and reverse: 5′-TTATCTGCAAGTCAGCGTAAC-3′ and for rs10887800 polymorphism forward: 5′-CAGGAAAGAAAAGAGTTGACAT-3′ and reverse: 5′-AAGTTGTTCCAGCTACTGT-3′. Genomic DNA (300 ng) was amplified in a final volume of 30 µl, containing: 10 mM TRIS–HCl buffer (pH 8.3), 50 mM KCl, 1.5 mM MgCl_2_, 200 µM each dNTP, 1 µM of each primer and 2 U Taq polymerase (all reagents from MBI Fermentas, St. Leon-Rot, Germany). Amplification of DNA was performed in a PTC 200 Thermal Cycler (MJ Research, Inc. Waltham, MA) with the initial denaturation at 95 °C for 6 min, followed by 35 cycles at 94 °C for 30 s, annealing at 60 °C for 30 s and extension at 72 °C for 1 min. The final extension step was at 72 °C for 7 min. The PCR products (10 µl) were digested for 6–10 h with 5 U of restriction endonucleases (Pst I for rs10887800 and Msp I for rs2576178 polymorphism) at 37 °C. The resulting DNA fragments were separated by electrophoresis on 2, 5 % agarose gel. After digestion with Pst I endonuclease, the PCR fragment sizes were: 554 bp (undigested) for A allele and 415 + 139 bp for a polymorphic variant (G allele). After digestion with Msp I, the fragment sizes were: 525 bp (undigested) for A allele and 423 bp + 102 bp in case of a polymorphic variant (G allele; both endonucleases from MBI Fermentas, Germany).

### Statistical analysis

Statistical analyses were performed using the IBM SPSS Statistics for Windows, version 20 (IBM Corp., Armonk, NY, USA). Baseline differences between two independent groups were evaluated by a Chi-square test of independence (for categorical variables) and a Student’s *t* test for means of continuous variables. Hardy–Weinberg equilibrium was assessed by a goodness-of-fit Chi-square test. Genotype distribution and allele frequencies were compared between groups using a 2 × 2 contingency Chi-square test. A multivariate logistic regression analysis was used to assess the genotypes impact on a CAD risk adjusted by age, gender, BMI, diabetes mellitus, hypertension, smoking, hyperlipidemia and stroke in anamnesis as potential confounders. Adjusted OR with 95 % confidence intervals (CI) was calculated. Each genotype was assessed under 2-d*f* codominant, 1-d*f* dominant and 1-d*f* recessive genetic models of inheritances. The wild-type homozygous group was the reference group for comparisons. All *p* values were two-sided and *p* < .05 was considered to indicate statistically significant differences.

## Results

Two renalase gene polymorphisms: rs10887800 (intron 6) and rs2576178 (5′ flanking region) were genotyped in 107 CAD+ patients and 202 CAD− patients. The baseline characteristics of the studied groups are summarized in Table 1.

**Table 1:** Demographic and clinical characteristics of studied groups

Variables	CAD+ (*n* = 107)	CAD− (*n* = 202)	*p* value
Age (years)	69.61 ± 10.51	61.20 ± 14.83	<.001
Sex (M/F)	64/43	93/109	<.001
Dialysis duration (years)	6.01 ± 6.16	6.52 ± 6.40	>.050
BMI (kg/m^2^)	27.47 ± 5.62	24.87 ± 4.93	<.001
Albumin (g/dl)	3.86 ± .36	3.98 ± .39	<.050
Hemoglobin (g/dl)	10.6 ± 1.3	11.00 ± 1.45	<.050
EPO demand (IU)/week	3841.58 ± 2704.04	3568.42 ± 2793.81	>.050
Diabetes mellitus (%)	48 (44.9)	41 (20.4)	<.050
Stroke in anamnesis (%)	13 (12.1)	12 (6.0)	>.050
Hyperlipidemia (%)	65 (61.3)	87 (43.5)	<.010
Family history of CVD	21 (19.6)	21 (10.4)	<.010
Smoking	21 (19.6)	27 (13.4)	>.050
Alcoholism	2 (1.9)	6 (3)	>.050
Rs10887800			
GG/AG/AA	32/51/24	39/110/53	.108
GG versus (AG + AA)	32/75	39/163	.035
G versus A	.54/.46	.47/.53	.088
Rs2576178			
GG/AG/AA	10/43/54	13/75/114	.490
G versus A	.29/.71	.25/.75	.251

Continuous variables are presented as mean ± SD. Discrete variables are presented as numbers and percentages (in parentheses)

*EPO* erythropoietin, *CVD* cardiovascular disorder, *CAD* coronary artery disease

The CAD+ patients were older (69.61 ± 10.51 vs 61.20 ± 14.83, *p* < .001) and more frequently male (59.8 vs 46 %, *p* < .001). As expected, compared to the CAD−, the CAD+ patients had a lower hemoglobin and albumin level and a higher prevalence of concomitant diseases, including diabetes mellitus and hyperlipidemia. Stroke in anamnesis was reported more often in the CAD+ group, but the difference was not statistically significant. The CAD+ patients were also more likely to have a history of cardiovascular disorders in a first-degree relative and a higher BMI mean value.

Genotype distribution and allele frequencies of rs10887800 and rs2576178 renalase gene polymorphisms were compared in the groups of patients with and without CAD. Genotyping results for these two polymorphisms are summarized in Table 1. The genotype distribution in all examined groups was in Hardy–Weinberg equilibrium.

For the rs10887800 polymorphism, no significant differences in allele frequencies were observed between groups. However, the frequency of the G allele in the CAD+ group was higher than in CAD− (.54 vs .47) and the difference showed a trend toward significance, OR 1.34 (95 % CI .96–1.86), *p* = .088. The analysis of the genotype distribution showed that there was significant difference in the frequency of GG genotype between CAD+ and CAD− groups [GG vs AG + AA], OR 1.78 (95 % CI 1.04–3.03), *p* = .035. The data were further examined by the logistic regression analysis for three models of genetic inheritance. After adjustment for age, sex, BMI, smoking status, hyperlipidemia, diabetes mellitus, hypertension and stroke in anamnesis, rs10887800GG genotype was associated with the increased risk of CAD under the codominant model [GG vs AA; adjusted OR 2.66 (95 % CI 1.19–5.94), *p* = .017] and under the recessive model [GG vs AG + AA; adjusted OR 2.10 (95 % CI 1.10–4.02), *p* = .025], Table 2.

**Table 2:** Results of multivariate logistic regression analysis of the rs10887800 polymorphism effect on CAD risk under three models of genetic inheritance

Rs10887800 A > G Genotypes	CAD+ *n* = 107	CAD− *n* = 202	CAD+ versus CAD−	
			OR^a^ (95 % CI)	*p*
Codominant model				
AA	24 (22.4)	53 (26.2)	Reference	–
AG	51 (47.7)	110 (54.5)	1.41 (.71–2.80)	.322
GG	32 (29.9)	39 (19.3)	2.66 (1.19–5.94)	.017
Dominant model				
AA	24 (22.4)	53 (26.2)	Reference	–
AG + GG	83 (77.6)	149 (73.8)	1.72 (.90–3.29)	.100
Recessive model				
AG + AA	75 (70.1)	163 (80.7)	Reference	–
GG	32 (29.9)	39 (19.3)	2.10 (1.10–4.02)	.025

Genotype distribution is presented as numbers (%). HWE test for CAD+ *χ* ^2^ = .18, *p* = .669, for CAD− *χ* ^2^ = 1.80, *p* = .180

*CAD* coronary artery disease

^a^Odds ratios adjusted for age, sex, smoking status, BMI hyperlipidemia, arterial hypertension, diabetes mellitus and stroke in anamnesis

For the rs2576178 polymorphism, no significant differences in allele and genotype frequencies were observed between groups. By multivariate analysis, the rs2576178 polymorphism did not influence the risk of CAD, Table 3.

**Table 3:** Results of multivariate logistic regression analysis of the rs2576178 polymorphism effect on CAD risk under three genetic models of inheritance

Rs2576178 A > G Genotypes	CAD+ *n* = 107	CAD− *n* = 202	CAD+ versus CAD−	
			OR^a^ (95 % CI)	*p*
Codominant model				
AA	54 (50.5)	114 (56.4)	Reference	–
AG	43 (40.2)	75 (37.1)	1.38 (.77–2.46)	.269
GG	10 (9.3)	13 (6.4)	2.10 (.67–6.60)	.202
Dominant model				
AA	54 (50.5)	114 (56.4)	Reference	–
AG + GG	53 (49.5)	88 (43.5)	1.46 (.84–2.55)	.174
Recessive model				
AG + AA	97 (90.7)	189 (93.5)	Reference	–
GG	10 (9.3)	13 (6.4)	1.83 (.60–5.58)	.286

Genotype distribution is presented as numbers (%). HWE test for CAD+ *χ* ^2^ = .11, *p* = .735, for CAD− *χ* ^2^ = .02, *p* = .888

*CAD* coronary artery disease

^a^Odds ratios adjusted for age, sex, smoking status, BMI hyperlipidemia, arterial hypertension, diabetes mellitus and stroke in anamnesis

## Discussion

In the present case–control study, we found that the rs10887800 renalase gene polymorphism significantly increased the risk of CAD in hemodialyzed patients. Moreover, the rs10887800 polymorphism affected the CAD risk independently of age, sex and other CAD risk factors, including smoking, BMI, hyperlipidemia, arterial hypertension and diabetes mellitus.

By multivariate logistic regression analysis, we found that under the codominant model patients with GG genotype had almost threefold higher risk of CAD compared to AA carriers. Under the recessive model, the risk of CAD was approximately twofold higher for G allele homozygotes than for A allele carriers.

As far as the authors know, no other papers concerning the association between renalase gene polymorphisms and CAD risk in ESRD patients have been published. In our previous study of 369 dialyzed patients, we found a link between two SNPs in the renalase gene and arterial hypertension. The G allele of both rs10887800 and rs2576178 polymorphisms was associated with an increased risk of hypertension (1.76 and 1.55 times, respectively) [12]. In another study of patients with chronic kidney disease from North India, the rs2576178 polymorphism did not affect the risk of hypertension. However, these authors observed that the CC genotype and the C allele of the rs2296545 renalase gene polymorphism were related to hypertensive nephropathy [13].

So far, a few studies have evaluated an association between SNPs in the renalase gene and CAD in patients with normal kidney function. The rs2576178 polymorphism was found to be involved in a CAD risk by Li et al. [14]. Contrary to our findings, these authors observed that Chinese hypertensive patients with either the AA genotype or the A allele were far more likely to develop CAD. The discrepancy between these results and our findings could be attributed to ethnic differences or different mechanisms for the effect of the polymorphism in the presence of chronic kidney disease. The effect could thus depend on the population studied. Li et al. did not observe any association between rs2296545 polymorphism and CAD risk. Consistent results were obtained by Fava et al. [15] of 5696 Swedes from a cardiovascular cohort of the “Malmö Diet and Cancer” study. After a follow-up period of 15 years, researchers did not find any association between rs2296545 polymorphism and CAD event developed in 408 subjects. In a study of Fava et al., rs2576178 polymorphism did not affect the CAD risk.

Farzaneh-Far et al. [16] in the study of 590 Caucasian individuals with stable CAD revealed that the CC genotype of rs2296545 polymorphism was associated with a left ventricular hypertrophy, systolic and diastolic dysfunction of the heart, poor exercise capacity and inducible ischemia.

The specific pathway by which rs10887800 polymorphism modified the risk of CAD remains unknown. The analyzed SNP is located near exon/intron boundary in a putative functional region, and thus, it might affect gene regulation and expression and lead to an altered amount of renalase produced. It was demonstrated in animal studies that deficiency of circulating renalase increased plasma catecholamine levels, decreased tolerance to ischemia and aggravated ischemic myocardial damage [17]. In another paper, renalase administration reduced myocardial damage during acute ischemia [18]. Zhou et al. [19] in the study of ApoE(–/–) mice suggested that renalase gene could be a potential-related gene of lipid metabolism and atherosclerosis. Furthermore, it was suspected that renalase could be the possible molecular target of valsartan to help stabilize atherosclerotic plaque. Thus, apart from the role of circulating renalase, the role of renalase expressed in a local cardiac tissue both in cardiomyocytes and in coronary arterial endothelium could be taken into consideration in the pathogenesis of CAD. Other unknown enzymatic functions of the renalase should be considered as well.

Human studies provided contradictory results. In the study of He et al. [20], plasma renalase level reversely correlated with the severity of the CAD in Chinese population, whereas Maciorowska et al. [21] observed an increased renalase level in the presence of CAD in hypertensive Caucasian individuals. Interestingly, Stojanovic et al. [22] have recently demonstrated that increased renalase concentration was a risk factor for reduced estimated glomerular filtration rate (eGFR < 60 ml/min/1.73 m^2^) in stable renal transplant recipients (RTR). Thus, the authors concluded that renalase could play a role in the development of late complications, including cardiovascular disorder after successful renal transplantation. Furthermore, since they observed higher renalase level in patients receiving cyclosporine compared to those on tacrolimus, they suggested that the activation of the sympathetic nervous system, rather than endothelial reduction of nitric oxide participated in the pathogenesis of cyclosporine-induced hypertension in RTR population.

Some limitations should be considered in the interpretation of our results. The study population involved only Caucasian individuals. Therefore, the results may not be generalized to other ethnic groups. Furthermore, serum renalase level or its activity which could be useful to clarify the obtained association was not determined. However, it will be the subject of ongoing studies.

## Conclusion

The present case–control study suggested for the first time that the rs10887800 renalase gene polymorphism may influence the susceptibility to CAD in hemodialyzed patients. The studied SNP could thus be considered as a novel genetic risk factor for CAD in hemodialyzed patients. Even though there are a lot of open questions, the presented results put an important value in understanding the mechanism of cardiovascular complications in patients with ESRD. Replication in a larger size sample and other ethnic groups are required to confirm the obtained association.
